# Effects of the Residential Environment on Health in Japan Linked with Travel Behavior

**DOI:** 10.3390/ijerph13020190

**Published:** 2016-02-03

**Authors:** David Perez Barbosa, Junyi Zhang, Hajime Seya

**Affiliations:** Graduate School for International Development and Cooperation, Hiroshima University, 1-5-1 Kagamiyama, Higashi-Hiroshima 739-8529, Japan; zjy@hiroshima-u.ac.jp (J.Z.); hseya@hiroshima-u.ac.jp (H.S.)

**Keywords:** active travel behavior, physical health, mental health, social health, residential environment

## Abstract

This paper aims to clarify how the residential environment is associated with overall health-related quality of life (QOL) via active travel (walking and cycling), by reflecting the influence of different trip purposes in Japan. The health-related QOL includes physical, mental, and social dimensions. For this study we implemented a questionnaire survey in 20 cities in Japan in 2010 and obtained valid answers from 1202 respondents. The residential environment is defined in terms of distances to and densities of different daily facilities extracted from both the survey and external GIS data. We found that the effects of residential environment on active travel behavior are mixed and limited, depending on types of trip makers. Unexpectedly, travel behavior has no direct effects on the health-related QOL. The residential environment, which is only observed indirectly via lifestyle habits for commuters, has limited effects on health. As for noncommuters, neither their travel behavior nor the residential environment influences their health-related QOL.

## 1. Introduction

It is generally accepted that the promotion of active travel (cycling and walking) in daily life can contribute to the improvement of health conditions, especially if cycling and walking replace short-distance car trips [1]. Travel is recognized as an essential component of life and a means of providing access to goods and services. Different travel modes are associated with specific impacts on society, including health, environment, and social effects [1]. On the other hand, the level of physical activity involved in daily travel is of particular interest because most people must travel to meet their daily life needs.

Most studies in the field of transportation have argued that travel is derived from participation in activities and the utility of travel is negative because of the necessity of investing time and/or money on it to be able to access the activity location [2,3,4,5]. For example, repeated long commute times usually bring tiredness to commuters, which may also influence their mental health. On the other hand, even if commuters just walk a short distance (e.g., 100 m) from home to the station, station to work (or another destination) and/or walk between stations, repeating it daily is surely a form of physical exercise, which may be beneficial to people’s physical health. Many health benefits can be derived as well from cycling, including good physical fitness and a longer lifespan. Similarly, walking or cycling during the commute to work may be associated with better mental health [6]. In a few specific cases, cycling may have adverse effects on body health [7], but in general, the beneficial effects of a regular cycling activity for both physical and mental health largely outweigh the detrimental effects [8]. Thus, encouraging people to walk and/or ride a bicycle more in their daily life is expected to increase their level of performing physical activities, which may have significant health benefits [7,8,9,10,11,12].

Among the benefits of walking and cycling for health, we can count a reduced risk of premature death, heart disease, diabetes, high blood pressure, breast cancer, depression and anxiety, and increased psychological well-being, among others [12,13,14,15,16,17,18,19]. The World Health Organization (WHO) European Charter on Counteracting Obesity mentions that safe cycling and walking are part of the package of measures and policies to be promoted to address overweight and obesity [14]. Among individual motivations to adopt cycling and walking as part of daily travel behavior, it is possible to count health, a desire to build community and familial ties, and financial considerations [20].

In Japan, urban cycling is a widely accepted transportation mode, even though Japanese cities do not have extensive cycling networks. Traditionally, Japanese urban cycle plans consider aspects such as shared pedestrian/bicycle circulation areas in most sidewalks, implementation of bicycle parking facilities around railway/subway stations and road markings for bicycle zones within road intersections [21]. It is important to note that under the current regulations, bicycles are not allowed on public transport in Japan, and a bicycle can only be carried under very restrictive conditions (the use of special bags to cover them is necessary, for instance).

While Japanese cities are amongst the largest and most populated in the world, residential neighborhoods within Japanese cities are largely self-contained in terms of services. Residents usually have to cycle no more than 5 to 10 min to reach supermarkets, kindergartens, schools, doctors, dentists, and other services covering most necessities for everyday living, since they can be found within walking or cycling distance without the need to travel excessive distances [22]. However, “self-contained” does not necessarily mean that residents are satisfied with their residential environment, living functions, and services provided. In fact, suburban large-scale shopping centers built along roadsides are popular in Japan. This is partially due to some dissatisfaction with the services provided by neighborhood shopping stores, and a car-dependent lifestyle. Other factors such as efficient public transport systems, the cost and inconvenience of owning a car in big metropolitan areas, and the provision of bicycle parking infrastructure are important factors that contribute to the extensive daily use of bicycles in almost all urban areas of Japan. In Table 1, we introduce the modal share information for some of the main cities of Japan that were included as survey locations in this study. As can be seen, nonmotorized trips in Japanese cities account for a very significant part of the modal share. While the use of cars can be as low as 13%, the use of nonmotorized modes (walking and cycling) for travel can be 28%–57% of all trips undertaken in the selected urban areas [23].

**Table 1:** Modal shares on weekdays in major Japanese cities.

City	Train	Bus	Car	Motorcycle	Bicycle	Walk & Others
Sapporo	17.6	3.9	42.0	0.3	11.4	24.8
Sendai	11.2	6.4	50.3	2.5	10.1	19.5
Saitama	30.1	1.7	26.6	1.8	18.4	21.4
Chiba	27.4	1.6	38.2	0.8	10.5	21.6
Tokyo (23 wards)	36.7	3.8	14.2	1.7	16.3	27.3
Yokohama	35.8	7.0	21.7	2.7	7.1	25.8
Kawasaki	42.0	3.5	15.7	1.7	12.1	25.0
Shizuoka	7.5	2.2	46.6	4.1	21.4	18.1
Nagoya	18.9	2.1	42.9	1.0	15.5	19.7
Kyoto	18.8	5.2	26.4	5.5	18.2	25.8
Osaka	30.0	2.2	13.6	2.2	27.6	24.3
Sakai	20.2	1.5	39.7	3.5	18.7	16.3
Kobe	27.6	4.6	29.5	3.3	9.1	25.8
Hiroshima	8.8	5.0	47.6	5.7	12.5	20.3
Kitakyusyu	5.2	8.0	56.9	1.9	5.8	22.2
Fukuoka	11.4	6.0	35.2	3.8	15.1	28.5

Source: The nationwide person-trip survey in Japan, 2010 [23].

Considering that health is seen as being more than just the absence of ill health, transport will have significant impacts, both positive and negative [24]. For most people, the easiest and most acceptable forms of physical activity are those that can be incorporated into everyday life, naturally including travel. Examples include walking or cycling instead of driving [24], something that we will refer to in this publication as active travel. However, we observe that while most of the literature on active travel and health still focuses on physical activity and obesity [19], other aspects of the relation between active travel and health are more difficult to find.

The purpose of this study is to provide additional evidence on how the residential environment affects use of active travel modes and health-related quality of life (QOL) in the context of Japan. Research questions we have formulated are as follows:

(1) Is the use of active travel modes associated with the residential environment?
(2) Does the use of active travel modes improve the health-related QOL?
(3) How does the residential environment affect the health-related QOL, considering the influence of active travel?

To answer these questions, we implemented a health-related QOL survey that included information relevant to these questions in major Japanese cities in 2010 and collected valid data from more than 1200 persons who represented the whole population with respect to age, gender, and residential locations (cities). Using the above data, we conducted a series of statistical analyses, including clustering analysis, ANOVA analysis, regression analysis, and structural equation modeling analysis. The remainder of this paper is organized as follows: Section 2 presents a brief literature review and Section 3 describes relevant concepts and measurement. Section 4 introduces data used in this study and Section 5 explains the statistical analysis results, together with discussions of policy implications. Finally, this study is concluded in Section 6.

## 2. Literature Review about Built Environment and Health

In this section, we discuss relevant findings from the existing literature on the characteristics of the built environment on the health-related QOL. More specifically, we discuss the influence of open public spaces, parks and other facilities on health, the relation between built environment and active travel, and the effects of urban density on active travel and health. 

The built environment, specifically the residential environment in this study, is considered to be one of many variables that affect physical activity [24]. It comprises mainly those urban design features that affect patterns of human activity within the environment [25]. Because this study focuses on the built environment surrounding residences, hereafter we use the term “residential environment.” 

### 2.1. The Influence of Open Spaces, Parks and Other Facilities

Many of the best places for increasing the activity levels are neither the home nor the workplace, but are rather “third places” in the public realm, such as streets, sidewalks, parks, cafes, theaters, and sports facilities. Such public places are important venues for a wide variety of activities, of which some—such as social interaction and physical activity—have clear health implications [26]. Those places create a sense of convenience and this convenience is often positively associated with walking [19], as are some aspects of urban design (particularly property density and street connectivity) [19,27]. Thus, these aspects need to be considered as a fundamental criterion when siting, designing, and building public places in ways that attract people, encourage them to socialize, and promote physical activity in the environment.

We know for a fact that having parks is beneficial for cities and urban dwellers. In city parks, people can spend time on activities, such as walking a dog, playing sports, eating outside, or enjoying the natural environment. Leisure activities in parks can provide many health benefits, from providing direct contact with nature and a cleaner environment, to offering opportunities for physical activity and social interaction [28]. A group of studies reviewed in the *American Journal of Preventive Medicine* showed that “creation of or enhanced access to places for physical activity combined with informational outreach” produced a 48.4% increase in the frequency of physical activity. The same studies showed that easy access to a place to exercise results in a 5.1% median increase in aerobic capacity, along with weight loss, a reduction in body fat, improvements in flexibility, and an increase in perceived energy [28,29]. Other studies have associated parks and their greenery with significantly higher levels of active travel and of not being overweight or obese, as well as with other self-rated health indicators that provide evidence for important causal pathways that could provide a focus for public health intervention strategies [30].

### 2.2. Built Environment and Active Travel Behavior

A built environment that promotes walking can be associated with improved health conditions in many different ways, so urban planners clearly need to integrate health and active living considerations fully into their work [24]. A positive relation was found between built environment factors (density of places of employment, household density, green and open spaces for recreation, number of street intersections) and walking activity at the neighborhood level [27]. 

Active transport, which includes travel by foot, bicycle, and other nonmotorized vehicles, has been identified as a strategy that could increase community physical activity levels while producing other environmental and social benefits. Access to large, attractive public open space increases the odds of higher levels of walking, and is said to be restorative, reducing mental fatigue, improving well-being, and increasing opportunities for social interaction [31]. The quality of the public realm and public spaces appears to be important for health, both mental and physical, yet further research is needed to quantify the strength of association between green spaces and urban health, but also to investigate the psycho-social and economic dimensions that are more difficult to measure [32]. 

### 2.3. The Effects of Density—Urban Sprawl and Agglomeration

Health research on the consequences of suburban sprawl has been to some extent limited [26]. We know that urban sprawl contributes to health inequalities because residents there have less access to exercise opportunities and healthy food than do others, usually wealthy people [31,33,34]. Sometimes, people want to live outside of city centers to avoid traffic congestion, noise, crime, and other problems, and to have homes with more square footage and yard space; however, there is substantial evidence that urban sprawl has negative effects on human health and the environment [26,31]. In general, more negative than positive effects of urban sprawl have been observed for public health, partially due to factors linked to physical activity, daily life, increased dependency on motorized travel, and reduced population densities.

Although most of the available evidence in the literature mentions the benefits to health of high density environments that encourage cycling and walking, some studies suggest the negative effects of high-density living on the availability of green areas where people can do healthy activities [35]. This might be applicable to the case of Japan. Research strongly suggests that greenery-filled public areas that are close to residences and easy to walk in should be further emphasized in the development and redevelopment in the metropolitan areas of Japan through cross-sectoral collaboration. Such greened areas positively influence the longevity of urban senior citizens, independent of attributes such as their age, sex, or socioeconomic status [36].

### 2.4. Final Observations

Attention to the health problems of the urban centers has focused largely on social and organizational factors rather than features of the built environment. Some studies in public health research suggest that environmental changes may be more effective in changing long-term physical activity patterns than are interventions centered on structured activities such as formal exercise programs. If so, then we may find that interventions to promote walking could contribute substantially toward increasing the activity levels of even the most sedentary residents [18].

Having enough evidence that the environment does influence levels of physical activity and obesity, another body of evidence appears to suggest that any influences of the environment are small, that the mechanisms by which environmental components may operate are as yet unclear, and that the exact environmental components that affect body weight and activity are yet to be identified [19]. Thus, further research is required to establish how different environments affect different individuals, because individuals interact with the environment on a number of levels, and experience effects from the physiological and emotional to those related to social, spiritual, and Intellectual well-being [37,38].

## 3. Health-Related Quality of Life

### 3.1. Concepts

The World Health Organization has noted that health is “a state of complete physical, mental, and social well-being and not merely an absence of disease and infirmity” [39]. *Physical health* assumes the ability to function normally in activities, including baseline activity (e.g., standing, walking slowly, and lifting lightweight objects) and health-enhancing physical activity (e.g., brisk walking, cycling, yoga, and dancing). The people who do only the baseline activity are considered to be physically unhealthy [40]. *Mental health* is a state of successful performance of mental function, resulting in productive activities, fulfilling relationships with other people, and the ability to adapt to change and to cope with challenges [40,41]. *Social health* relates to one’s ability to participate in society, fulfilling roles as family member, friend, worker, or citizen or in other ways engaging in interactions with others [40,42].

### 3.2. Measurement

The 36-item Short Form (SF-36) is the most commonly used instrument for measuring health-related QOL [43,44,45,46]. It consists of 36 items that present respondents with choices about their perception of their own health condition and yields eight health subscales, as follows:

- Physical functioning (PF): Feeling limitations in performing physical activities;
- Role-physical (RP): Experiencing problems with work or other daily activities as a result of physical health;
- Bodily pain (BP): Experiencing physical pains, or limitations due to physical pains;
- General health (GH): Feeling in poor, fair or excellent health;
- Vitality (VT): Feeling tired, worn out, or full of pep and energy;
- Social functioning (SF): Having extreme, frequent, or no interference with normal social activities due to physical and emotional problems;
- Role-emotional (RE): Experiencing problems with work or other daily activities as a result of emotional problems;
- Mental health (MH): Feeling nervous, depressed, calm, peaceful, or happy.

**Figure 1:**
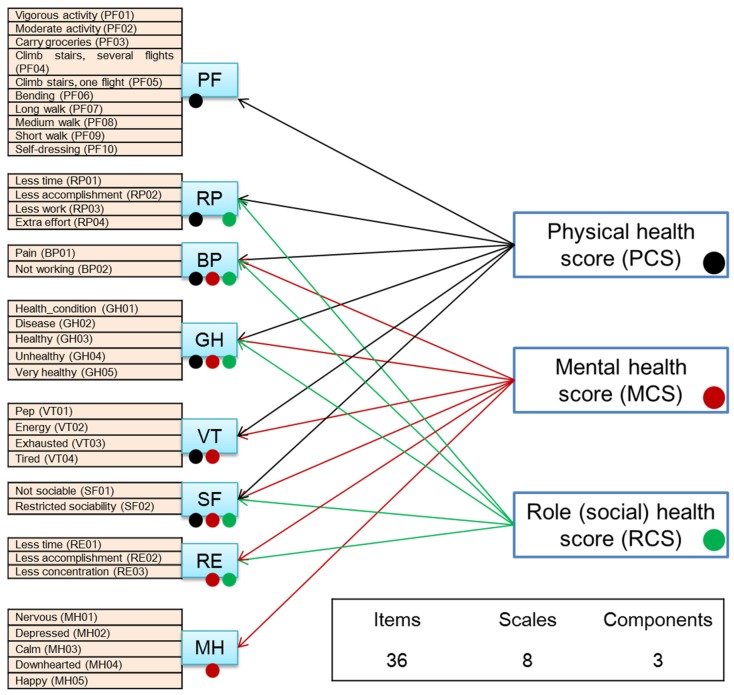
Scales and components in the SF-36 model.

After the items were grouped into the aforementioned eight subscales, three different health component scores were computed: physical (PCS), mental (MCS), and role-social (RCS). These definitions were adopted from the model used by Suzukamo *et al*. [35], whose findings support the use of a three-component model of SF-36 in Japan, after successful validation testing (see Figure 1).

Based on the items’ and subscales’ individual answers, the different health component scores PCS, MCS, and RCS were calculated for each individual [43,44,45,47].

There is a large body of evidence showing obesity as an important outcome of a poor health condition and relating the effects of the residential environment to people’s obesity [19,37,38,48,49,50]. By using the body mass index as a key indicator, clear associations between obesity and population density and other environmental features have been demonstrated [38,48,50]. Despite the relative prevalence of studies focusing on the physical dimensions of health, a growing body of literature is making associations between the urban environment and health in its mental and social dimensions [31,51,52].

As a part of the SF-36 questionnaire, we can find items that are directly associated with time spent in active travel and active lifestyle activities, which include taking part in sports, walking for short and long distances, and participating in social activities with friends and relatives out of the home, *etc*. Therefore, some measurement items of the SF-36 can be directly influenced by an active travel behavior. On the other hand, the emotional experiences felt during a trip can play a very important role in enhancing or worsening the general health-related QOL and individual well-being, particularly if those trips are done on a regular basis. Studies have found how the relation between transport and health-related QOL can have significant associations, contributing to enhancing the health-related QOL, improving personal well-being, and reducing the risk of social exclusion [53,54,55].

## 4. Data 

A health-related QOL survey was implemented in 20 major Japanese cities during 22–29 November 2010. The selected areas were Tokyo, Osaka, and Nagoya (as the three megacity metropolitan areas in Japan), and 17 other government-ordinance-designated cities that are smaller in terms of population but still with a population larger than 500,000 inhabitants: Sapporo, Sendai, Saitama, Chiba, Yokohama, Kawasaki, Sagamihara, Niigata, Shizuoka, Hamamatsu, Kyoto, Sakai, Kobe, Okayama, Hiroshima, Kitakyushu, and Fukuoka.

For this questionnaire survey, the target number of samples was set to 1000 persons. The survey was done with the help of a major Internet survey company, which had more than 1.4 million registered members. The respondents were randomly selected but they reflected some representative attributes of the population (age, gender, and residential locations). To reach the desired target within a limited survey time (a week), a total of 14,534 members were contacted. As a result, valid answers were successfully collected from 1,213 persons, with a return rate of 8.3% [45].

Additionally, ArcGIS (Esri Inc., Redlands, CA, USA) was used to develop residential environment measures by using the postcode of residential location, with a total of 1199 individuals whose corresponding postcode was a valid match for the GIS information. We consider that the sample size (*N* = 1199) is adequate for our research purposes, as it is balanced and representative of the characteristics of population in Japanese cities, in terms of age, gender, income, and other attributes as well.

### 4.1. GIS Data Matching

Based on the postcode of residential location, it is possible to obtain land-use-related information of those locations from the National Land Numerical Information [56] service provided by the Ministry of Land, Infrastructure, Transport and Tourism of Japan (MLIT). The National Land Numerical Information is digitized geographic information on topography, land use, public facilities, roads, and railroads, and other land-related information. Grid cell (meshed) data comprise much of the data that can be combined with population and other statistical data to conduct further analyses. The land-use-related information used in this study consists of:

• Land-related attributes of the zone: Based on the postcode area, we determined whether the predominant land use in a location is commercial or not as an indicator of mixed land use. Similarly, we can determine a value for the population in the corresponding cell of the mesh data. Each cell has an area of 1 km^2^, so this value is equivalent to the population density of the corresponding residential location [57]. The last year of updated information for the predominant type of land use in each cell of the mesh was 2011, and for the population density it was 2010.
• Park location: Based on park locations, we can calculate corresponding measures of distance and number of parks within a 1 km radius of each resident’s residential location. The last updated information for this layer is from 2011, and relates to the built parks based on the Urban Park Act of Japan.
• Cultural facilities: Museums, libraries, memorial halls, and other cultural facilities in the zone are included as cultural facilities. Similarly, we can calculate the corresponding measures for the distance and number of cultural facilities. The most recent collection of data for this information was in 2012.

Since we face a limitation of not knowing the exact residential location, and because of considerations with regard to the privacy of the respondents, we can only rely on the postcode rather than the address. However, using postcodes, we can identify the address not only at a municipality but also at a district level, with small sizes that comprise walkable distances. This enables us to combine spatially fine existing grid data sets (of around 1 km^2^) provided by government bodies for our analysis. This means that distance measures (to city parks and cultural facilities) may suffer unavoidable measurement error bias to a certain degree when we use the center of a district to approximate actual housing/condominium locations. In addition, inaccurate and incomplete data may cloud the validity of GIS-based measures and stem from multiple factors [58]. For our research purposes, we consider the GIS-based measures to be valid, as they can reflect with sufficient accuracy the characteristics of the residential environment that we aimed to study.

### 4.2. Survey Contents

The questionnaire consists of questions about travel behavior, health-related QOL, residential environment, lifestyle habit, health promotion activities, park usage, QOL (happiness and life satisfaction), and individual and household attributes. Here, only travel behavior, health-related QOL, and residential environment are described in detail. Definitions of the different variables are listed in Table 2.

### 4.3. Travel Behavior

The travel behavior of the individuals is characterized in terms of the activity (purpose of travel), the frequency, the travel mode, and the moving distance. For activity, 11 different purposes of travel are listed: commuting, doing other business, shopping, pursuing leisure activities, doing sports, engaging in nonacademic learning, pursuing social activities, attending to health care, eating out, taking care of personal matters, and others. In the questionnaire, the frequency of travel is characterized by using an ordinal scale from 0 to 10, in which 0 is assigned when the activity is not considered by the respondent, 1 is equivalent to a few times a year, and 10 is equivalent to an daily/almost daily activity. The travel modes considered in the study were walking, riding a bicycle, riding a motorcycle, using a car (as the driver), using a car (as a passenger), taking a train, taking a streetcar, taking a monorail, taking a bus, taking a taxi, and others. For the purposes of this study, we group the walkers and cyclists as active travelers. We characterize the active travel behavior by calculating a joint index, *i.e.*, Active Travel Score (ATS), to describe the use of active travel modes and the frequency, as follows:

(1) $$ATS_{n}=\sum^{\text{​}}f_{ni}a_{ni}$$

Here, *f_ni_* is individual *n*’s frequency for travel by purpose (activity) *i* (where *i* =1, 2,...,11) and *a_ni_* is a dummy variable indicating whether an active travel mode is used by individual *n* for travel purpose *i*. Thus, the ATS indicates roughly how much or how often an individual uses active travel modes in comparison to other people. The score for an individual who walks or rides a bicycle to engage in any of the aforementioned activities is higher than those who do not, and the more frequent an individual walks or rides a bicycle, the higher the score is.

**Table 2:** Variables selected for this study.

Category	Description	Min	Max	Mean	SD
Residential environment					
No. of parks	Number of parks within a 1 km radius from the residential location	0	68	18.39	9.84
Distance to park	Distance to the closest park from the residential location (m)	14.2	2656.6	222.2	182.9
No. of cultural facilities	Number of cultural facilities within a 1 km radius from the residential location	0	31	5.64	5.27
Distance to cultural facility	Distance to the closest cultural facility from the residential location (m)	8.1	2929.4	590.2	379.51
Population density	Number of inhabitants in the corresponding 1 km^2^ area of the residential location	127	28,738	10,984.4	6026.18
Commercial land use	Dummy variable: 1 if the use of land is predominantly commercial, 0 otherwise.	0	1	0.19	0.39
Health-related QOL					
PCS	Physical Component Score	5.4	100	73.43	14.81
MCS	Mental Component Score	3.2	100	69.14	15.86
RCS	Role (Social) Component Score	0.0	100	79.78	16.41
Individual attributes					
Age	Age in years	15	69	42.12	13.39
Gender	1 if male, 0 if female	0	1	0.50	0.50
Driving license ownership	1 if there is possession, 0 otherwise	0	1	0.84	0.37
Car ownership	1 if there is possession, 0 otherwise	0	1	0.50	0.50
Household size	Number of household members	1	9	2.75	1.30
Lifestyle habits					
Breakfast	Eat breakfast everyday (1: Rarely, 5: Everyday)	1	5	4.18	1.20
Sleep	Sleep 7–8 h (1: Rarely, 5: Everyday)	1	5	3.24	1.27
Meal	Meal is balanced/nutritious (1: Rarely, 5: Everyday)	1	5	3.47	1.01
Smoke	Do not smoke (1: Rarely, 5: Everyday)	1	5	4.16	1.55
Sports	Practice sports periodically (1: Rarely, 5: Everyday)	1	5	2.71	1.38
Alcohol	Do not drink much alcohol (1: Rarely, 5: Everyday)	1	5	4.14	1.19
Work	Work within 9 h a day (1: Rarely, 5: Everyday)	1	5	3.43	1.40
Stress	Do not feel much conscious stress (1: Rarely, 5: Everyday)	1	5	3.00	1.18
Health-related QOL scales					
General health	Calculated value for the corresponding health scale	0	100	58.33	19.05
Physical functioning	Calculated value for the corresponding health scale	0	100	91.24	14.19
Role-physical	Calculated value for the corresponding health scale	0	100	87.87	20.66
Role-emotional	Calculated value for the corresponding health scale	0	100	85.73	21.92
Social functioning	Calculated value for the corresponding health scale	0	100	82.09	22.66
Bodily pain	Calculated value for the corresponding health scale	0	100	77.53	21.40
Vitality	Calculated value for the corresponding health scale	0	100	54.86	20.24
Mental health	Calculated value for the corresponding health scale	0	100	64.57	19.73
Travel behavior					
Walking	Active Travel Score (ATS) for walking	0	95	11.59	14.31
Cycling	Active Travel Score (ATS) for cycling	0	79	7.38	13.21
Public Transport	Equivalent score for the use of public transport	0	73	6.35	10.69
Active commuting	Commuting by active travel modes	0	10	2.22	4.12
PT commuting	Commuting by public transport	0	10	2.42	4.26
Active NC travel	Active modes by non-commuting purpose	0	85	16.76	15.91
PT travel	Public transport by non-commuting purpose	0	63	3.93	8.22
Travel purpose					
Frequency by purpose	Numerical scale equivalent to the number of days in a week (see analysis in Table 3)	0	5		

### 4.4. Health-Related QOL

According to the conceptual definitions of the SF-36 model, questions aiming to obtain scores for the 36 different items are included in the survey questionnaire. These questions include: ability to perform baseline activity, and perceptions of individual health condition, mental condition, well-being, accomplishments, and possible limitations in daily activities due to health limitations, as explained previously [43,44].

### 4.5. Residential Environment

Characteristics of the residential environment included in the analysis were obtained from both the questionnaire survey and GIS data. The influence of parks, cultural facilities, commercial facilities, and population density in the health-related QOL and the active travel behavior is examined.

### 4.6. Individual Attributes

Age, gender, driving license, car possession, occupation, income, and household characteristics are included here.

## 5. Results of Statistical Analyses

**Figure 2:**
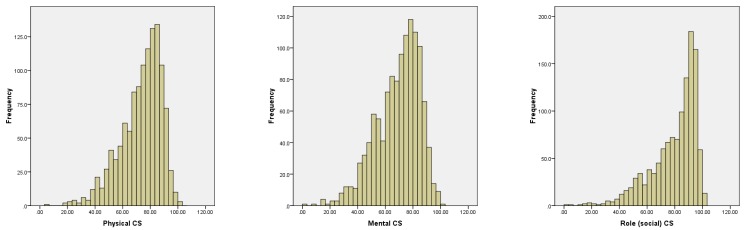
Histogram for the health related indicators (PCS, MCS and RCS).

In the questionnaire survey we asked the respondents about their travel frequency for different purposes. Only commuting behavior revealed important information, but the observed heterogeneity with respect to the other purposes of travel makes the overall travel behavior more difficult to grasp. In order to make a more simplified travel behavior analysis, we use cluster analysis techniques to find travel behavior patterns that are sufficiently representative to make clusters of individuals with similar travel behavior characteristics. After grouping the individuals in their respective clusters, we employ regression methods and structural equation modeling to analyze the direct and indirect effects of the residential environment on the active travel behavior and the health-related QOL in each of those groups. Histograms for the health-related QOL scales among respondents in the sample are shown in Figure 2.

### 5.1. Segmenting Travel Behavior

Here, we made use of hierarchical cluster analysis to classify individuals so as to capture the influence of heterogeneity based on the variation of the frequency by travel purpose. We found an acceptable solution was to divide the sample into three clusters to group the respondents by their frequency. Then we employed the Euclidean distance method for minimization of the distance to each centroid cluster, and each individual was assigned to one of clusters 1, 2, or 3 (containing 312, 501, and 386 individuals, respectively). Table 3 shows the cross-tabulation results for the travel frequency average values by group, the standard deviation in parentheses, the travel frequency values that define the cluster centers, and the number (and percentage) of participants that take part in each travel purpose, respectively. For the cluster analysis, the frequencies are converted into an equivalent numerical scale that reflects how many days per week the respondent travels to take part in each activity (see Table 3).

As defined by the cluster center values, the individuals in Cluster 1 can be characterized by the almost daily average frequencies for commuting and daily travel frequencies for business purposes, and occasional frequencies for other activities such as leisure, sports, and eating out. The individuals in Cluster 2 do not commute, but travel occasionally to go shopping, or to take part in other activities related to sports, health care, or eating out. The individuals in Cluster 3 are daily commuters who occasionally travel to go shopping, but in general, they make trips for different purposes less frequently than do individuals in Cluster 1. 

### 5.2. Features of Different Clusters

Table 4 shows how the identified three clusters listed in Table 3 are different from each other. ANOVA analyses were conducted with respect to the information collected in the survey, including individual attributes, residential environment, health-related QOL, lifestyle habits, and travel behavior.

Cluster 1 is a group of active members. In this cluster, 71% of the group members are male, and people in this group have the smallest average household size. Despite having higher numbers of people with driving licenses and higher levels of car ownership than people in Clusters 2 and 3, these individuals have the most active lifestyles, reflected by their travel habits and in their ATS scores for the use of cycling, walking, and public transport, far exceeding those for members of Clusters 2 and 3; and their use of active modes and of public transport for noncommuting purposes is significantly much higher than for those in Clusters 2 and 3. However, individuals in this group do not use active travel modes for commuting significantly more than individuals in Cluster 3.

**Table 3:** Results of cluster analysis.

Travel Purpose	Clusters (Average Travel Frequency (Standard Deviation))			Cluster Centers for Travel Frequency			Respondents in the Entire Sample
	Cluster 1	Cluster 2	Cluster 3	Cluster 1	Cluster 2	Cluster 3	
Commuting	4.71 (1.14)	0.23 (0.50)	5.00 (0.00)	4.7	0.2	5.0	750 (62.4%)
Business	5.00 (0.00)	0.20 (0.79)	0.07 (0.35)	5.0	0.2	0.1	388 (32.2%)
Shopping	1.93 (1.71)	2.48 (1.83)	1.56 (1.61)	1.9	2.5	1.6	1006 (83.5%)
Leisure	0.77 (1.11)	0.56 (1.03)	0.60 (1.03)	0.8	0.6	0.6	690 (57.0%)
Sports	0.73 (1.33)	0.95 (1.62)	0.61 (1.24)	0.7	1.0	0.6	531 (44.1%)
Non-academic learning	0.26 (0.96)	0.12 (0.64)	0.18 (0.79)	0.3	0.1	0.2	120 (9.9%)
Social activities	0.06 (0.32)	0.14 (0.50)	0.04 (0.21)	0.1	0.1	0.0	197 (16.4%)
Health care	0.18 (0.62)	0.18 (0.45)	0.076 (0.21)	0.2	0.2	0.1	484 (40.1%)
Eating out	0.71 (1.15)	0.36 (0.67)	0.50 (1.02)	0.7	0.4	0.5	685 (56.8%)
Personal affairs	0.28 (0.62)	0.26 (0.37)	0.12 (0.35)	0.3	0.3	0.1	677 (56.1%)
Others	0.44 (1.14)	0.31 (0.86)	0.17 (0.65)	0.4	0.3	0.2	331 (27.4%)
Number of individuals (N)	312	501	386				1199

Note: Values of frequency are represented in equivalent days in a week.

Cluster 2 can be defined as the less mobile group. Individuals in this group do not commute, but they make use of walking and cycling trips that are associated mainly with shopping, and occasionally with leisure, health care or other personal matters. This is group in which 71% of the respondents are women, and individuals in this group are the oldest among the respondents. They live in areas with higher concentrations of parks in their surroundings. On the other hand, they tend to have higher values in the categories of vitality and mental health. 

**Table 4:** ANOVA analysis results.

Variables	Cluster 1	Cluster 2	Cluster 3	*F* Value	Significance Level
**Residential environment**					
No. of parks	17.45	19.37	17.89	4.42	0.012
Distance to park	215.2	219.39	235.0	1.21	0.299
No. of cultural facilities	6.36	5.14	5.71	5.26	0.005
Distance to cultural facility	580.32	615.05	566.23	1.95	0.143
Population density	11551	10538	11104	2.82	0.060
Commercial land use	0.23	0.17	0.17	2.37	0.094
**Health-related QOL attributes**					
PCS	73.1	72.8	74.4	1.31	0.027
MCS	68.4	69.7	69.0	0.68	0.507
RCS	80.2	78.3	81.4	4.09	0.017
**Health-related scales**					
General health	57.1	58.2	59.4	1.23	0.293
Physical functioning	92.5	88.2	94.1	21.65	0.000
Role-physical	88.9	84.7	91.1	11.22	0.000
Role-emotional	86.8	84.1	86.9	2.36	0.095
Social functioning	82.5	81.4	86.9	0.35	0.707
Bodily pain	77.4	76.1	79.5	2.86	0.057
Vitality	52.5	58.1	52.5	11.5	0.000
Mental health	63.1	66.8	62.8	5.8	0.003
**Individual attributes**					
Age	41.8	47.3	35.7	93.6	0.000
Gender	0.71	0.29	0.61	89.9	0.000
Driving license ownership	0.90	0.82	0.81	6.21	0.002
Car ownership	0.59	0.48	0.45	6.98	0.001
Household size	2.62	2.87	2.70	3.756	0.024
**Lifestyle habits**					
Breakfast	3.97	4.40	4.06	15.6	0.000
Sleep	2.95	3.61	3.00	38.7	0.000
Meal	3.31	3.79	3.19	46.6	0.000
Smoke	3.76	4.33	4.26	14.6	0.000
Sports	2.70	2.76	2.67	0.48	0.617
Alcohol	3.91	4.31	4.11	11.6	0.000
Work	3.13	3.63	3.40	12.64	0.000
Stress	2.79	3.23	2.86	17.5	0.000
**Travel behavior**					
Walking	16.04	11.53	8.0	28.6	0.000
Cycling	8.04	6.88	7.49	0.76	0.467
Public Transport	9.87	3.20	7.55	44.2	0.000
Active commuting	2.79	0.49	3.99	96.2	0.000
PT commuting	4.07	0.40	3.69	115.1	0.000
Active NC travel	21.3	17.9	11.5	37.3	0.000
PT travel	5.80	2.79	3.87	13.2	0.000

Cluster 3 can be called the commuters-only group. The mobility patterns of individuals belonging to this group are mostly associated with commuting and shopping errands on an occasional basis. Regarding their active travel behavior, based on their ATS scores, we can observe that they use active modes less frequently in comparison with individuals in Clusters 1 and 2. We can observe that they travel mostly for their commuting needs, and their lifestyle is less active than the lifestyle of people in Cluster 1, since they travel less frequently for purposes other than commuting in general.

### 5.3. Direct Effects of Residential Environment on Active Travel Behavior

To analyze the distribution of ATSs, we make use of binary logistic and Tobit regression models. These models are used to examine whether and how different elements of the residential environment affect the active travel behavior, *i.e*., the use of cycling and walking for commuting and other travel purposes. In total, four regression models are estimated with respect to the following four dependent variables defined by the ATS (see Table 5):

(1) Active travel by walking
(2) Active travel by cycling
(3) Commuting by active travel modes (walking and cycling)
(4) Noncommuting activity by active travel modes (walking and cycling)

It is important to mention that for “(3) commuting by active travel modes,” we employed a binary logistic regression instead of a Tobit regression. The reason is, by nature of its own definition, the (active) commuting will adopt either the minimum or the maximum frequency values only (*i.e*., “0” or “10”), but no intermediate values in the frequency scale, thereby reducing the dependent variable to two possible outcomes that are transformed into a binary-equivalent code to employ the binary logistic regression model.

Here, distance to the closest park, distance to the closest cultural facility, number of parks within a 1 km radius of residence, number of cultural facilities within a 1 km radius of residence, commercial land use, and population density are used as explanatory variables. Major observations are summarized below.

(1) Parks are not influential to the use of active travel for any purposes, except for cycling activities in Cluster 1. Generally speaking, as shown in Table 2, parks are located within walking distance of the home. But this does not necessarily mean that people like to visit parks frequently. In reality, those parks nearby residence are usually very small and they are not suitable places in which people can do physical exercise. Instead, what is often observed is that some married women sometimes visit those parks with their children, and children of elementary and/or secondary schools play in those parks. One or more large-scale parks are found in many cities, but these are normally far from residences. These facts may support this finding.
(2) Cultural facilities are found to affect active travel behavior. The closer the distance to the nearest cultural facility, the higher the walking frequency for Clusters 1 and 2, the more use of active commuting for Cluster 3, the more noncommuting active travel for Cluster 2, and the more cycling for Cluster 3. The higher the number of cultural facilities near the residence, the more noncommuting activities by active travel modes can be observed for Cluster 1. If there are larger numbers of cultural facilities nearby the residence, people belonging to Cluster 1 are more likely to use active travel modes.
(3) If commercial land use is predominant in a residential location, the residents in all clusters are more likely to do active travel, both for commuting and noncommuting purposes, and individuals in Clusters 2 and 3 will generally prefer to walk more. For cycling activities, no influence of a commercial or mixed land use was observed.
(4) Population density is positively associated with the use of active travel for noncommuting purposes in all the clusters, relevant to use of active travel for commuting in Cluster 3, relevant for cycling in Cluster 2, and for more walking activities among commuters (individuals in Clusters 1 and 3). Increased population density within residential areas will increase the use of walking for the commuters and for the noncommuting activities by active travel modes for people in general.

**Table 5:** Direct effects of residential environment on active travel behavior.

Clusters	Cluster 1		Cluster 2		Cluster 3	
Statistical Values	Coefficient	*p*-Value	Coefficient	*p*-Value	Coefficient	*p*-Value
Active Travel by Walking						
Constant term	11.35		5.40		1.487	
Distance to park	0.004	0.636	−0.0004	0.935	−0.0003	0.963
Distance to cultural facility	−**0.0115**	**0.008**	−**0.00048**	**0.090**	−0.0036	0.324
No. of parks	−0.013	0.936	−0.0075	0.943	−0.154	0.229
No. of cultural facilities	−0.355	0.602	0.0451	0.935	−0.504	0.423
Commercial land use	1.13	0.721	**9.28**	**0.001**	**4.569**	**0.100**
Population density	**0.00058**	**0.016**	0.00017	0.307	**0.00035**	**0.055**
Log likelihood	−1070.19		−1497.45		−971.95	
Pseudo R-squared	0.0101		0.0085		0.0052	
**Active Travel by Cycling**						
Constant term	−5.32		−14.58		−14.92	
Distance to park	−0.0027	0.874	−0.0046	0.625	0.192	0.106
Distance to cultural facility	−0.011	0.181	−0.0076	0.111	−**0.012**	**0.092**
No. of parks	−**0.615**	**0.076**	0.191	0.256	0.183	0.402
No. of cultural facilities	−**2.38**	**0.095**	−**1.574**	**0.091**	0.652	0.514
Commercial land use	2.43	0.691	−6.085	0.152	1.719	0.722
Population density	0.0007	0.115	**0.0014**	**0.000**	0.00012	0.693
Log likelihood	−592.91		−951.6		−774.83	
Pseudo R-squared	0.0078		0.0176		0.0064	
**Commuting by Active Travel Modes (Walking and Cycling)**						
Constant term	−0.327				0.1984	
Distance to park	−0.00036	0.675			0.0004	0.541
Distance to cultural facility	−0.0005	0.255			−**0.0009**	**0.025**
No. of parks	−0.019	0.245			0.0042	0.743
No. of cultural facilities	−0.289	0.724			−0.0068	0.911
Commercial land use	**0.732**	**0.013**			**0.579**	**0.041**
Population density	−0.000005	0.830			−**0.00003**	**0.084**
Log likelihood	−179.77				−253.34	
R-squared	0.0264				0.056	
**Non-commuting Activity by Active Travel Modes (Walking and Cycling)**						
Constant term	18.46		11.86		3.37	
Distance to park	0.010	0.101	−0.0026	0.596	0.007	0.236
Distance to cultural facility	−0.004	0.171	−**0.0055**	**0.030**	−0.0053	0.102
No. of parks	0.031	0.756	−0.0633	0.511	0.0030	0.978
No. of cultural facilities	**0.339**	**0.054**	−0.3538	0.484	−0.0439	0.933
Commercial land use	**6.203**	**0.006**	**5.239**	**0.032**	**4.219**	**0.081**
Population density	**0.00043**	**0.007**	**0.00076**	**0.000**	**0.00042**	**0.009**
Log likelihood	−1176.60		−1777.54		−1171.785	
R-squared	0.015		0.0139		0.0080	

Note: figures in **bond** type mean they are statistically significant at the 1%, 5%, or 10% level. Note 2: For commuting by active travel modes, binary logistic regression was used instead of a Tobit regression. Note 3: Individuals in *Cluster 2* are non-commuters.

In summary, with respect to the first research question, the residential environment has marked effects on the use of active travel, but effects differ across different types of trip makers. The effects are not only mixed depending on types of trip makers but they also very limited because, in many cases, significant effects are only observed with respect to one or two elements of the residential environment. 

### 5.4. Effects of Residential Environment on Health Associated with Active Travel

**Figure 3:**
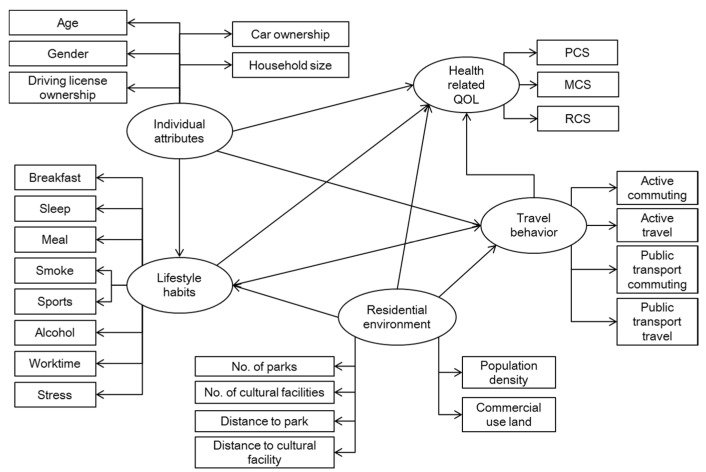
The basic SEM model assumed in this study.

To answer the second and third research questions in light of the above regression results, it is better to look at both questions within the same analysis framework. In other words, conditional on the above regression observations, we need a framework to jointly accommodate the relationships between residential environment and active travel, between active travel and the health-related QOL, and between residential environment and the health-related QOL. To this end, we build a structural equation model (SEM) with latent variables, as shown in Figure 3, which includes the following latent variables.

• *Residential environment*: the same set of variables as in the above regression analyses is selected to represent the residential environment.
• *Health-related QOL*: PCS, MCS, and RCS scores are the observed variables.
• *Lifestyle habits:* eight habits—regular exercise, alcohol consumption, smoking, sleeping patterns, nutritional balance, breakfast, working pattern, and subjective stress [59]—are measured in terms of the frequency of practicing these habits.
• *Active travel*: this is based on the definition of the Active Travel Score (ATS) and the observed travel frequencies by purpose and by mode. We calculated four different scores for commuting by active travel, noncommuting activity by active travel, commuting by public transport, and noncommuting travel by public transport. The first two scores are the same as in the above regression analyses. The public transport scores are measured by frequencies of using buses, trains, trams, or subways.
• *Personal attributes:* these include age, gender, ownership of driving license, ownership of vehicle, and household size. It is assumed that these personal attributes may influence all other four latent variables.

**Figure 4:**
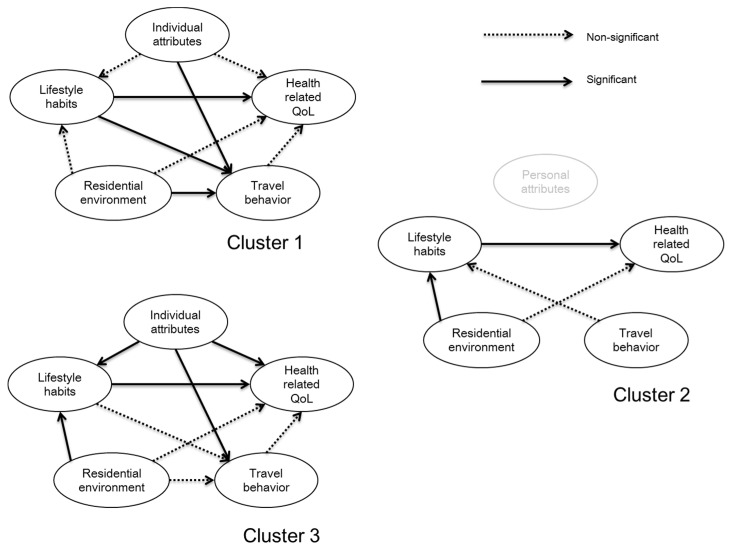
Variations in SEM model structures by clusters.

In short, it is assumed here that the residential environment may have both direct and indirect effects on the health-related QOL, where the indirect effects are observed via the practice of lifestyle habits and active travel. Modeling estimation results are shown in Figure 4 and Table 6. In Figure 4, the dashed lines represent the nonsignificant paths of influence in the SEM model and the solid lines represent the significant causal relationships (paths) that have been found in the different model estimations. 

**Table 6:** Results of the SEM model by clusters.

Variables	Cluster 1		Cluster 2		Cluster 3	
	Standardized Parameter	*p*-Value	Standardized Parameter	*p*-Value	Standardized Parameter	*p*-Value
**Individual attributes** *to explain the following endogenous latent variables*						
Lifestyle habits	0.008	0.930			**−0.162**	**0.041**
Travel behavior	**−0.352**	**0.001**			**−0.389**	**0.002**
Health-related QOL	0.117	0.148			**0.19**	**0.008**
**Residential environment** *to explain the following endogenous latent variables*						
Lifestyle habits	0.035	0.667	**−0.233**	**0.000**	**−0.148**	**0.063**
Travel behavior	**0.288**	**0.000**			0.068	0.440
Health-related QOL	**−**0.042	0.533	**−**0.068	0.224	0.017	0.769
**Lifestyle habits** *to explain the following endogenous latent variables*						
Health-related QOL	**0.493**	**0.000**	**0.28**	**0.000**	**0.636**	**0.000**
Travel behavior	**0.240**	**0.001**			0.158	0.117
**Travel behavior** *to explain the following endogenous latent variables*						
Lifestyle habits	0.335	0.014	0.947	0.560		
Health-related QOL	**−**0.057	0.458			**−**0.064	0.445
**Individual attributes** *to explain the following exogenous observed variables*						
Age	0.286				0.6	
Gender	**0.440**	**0.000**			**0.251**	**0.000**
Driving license ownership	**0.529**	**0.000**			**0.672**	**0.000**
Car ownership	**0.694**	**0.000**			**0.665**	**0.000**
Household size	**0.167**	**0.038**			**−**0.1	0.102
**Lifestyle habits** *to explain the following exogenous observed variables*						
Breakfast	**0.364**	**0.000**	**0.546**	**0.000**	**0.28**	**0.000**
Sleep	**0.523**	**0.000**	**0.362**	**0.000**	**0.321**	**0.000**
Meal	**0.636**	**0.000**	**0.704**	**0.000**	**0.379**	**0.000**
Smoke	**0.188**	**0.007**	**0.346**	**0.000**	**0.202**	**0.001**
Sports	**0.343**	**0.000**	**0.483**	**0.000**	**0.397**	**0.000**
Alcohol	0.110	0.108	**0.22**	**0.000**	**0.137**	**0.027**
Work	**0.436**	**0.000**	**0.26**	**0.000**	**0.389**	**0.000**
Stress	0.546		0.504		0.36	
**Health-related QOL** *to explain the following exogenous observed variables*						
PCS	0.999		0.994		0.998	
MCS	**0.952**	**0.000**	**0.958**	**0.000**	**0.965**	**0.000**
RCS	**0.927**	**0.000**	**0.951**	**0.000**	**0.938**	**0.000**
**Residential environment** *to explain the following exogenous observed variables*						
No. of parks	**0.267**	**0.000**	**0.14**	**0.013**	**0.743**	**0.000**
Distance to park	**−0.229**	**0.001**	**−0.171**	**0.003**	**−0.625**	**0.000**
No. of cultural facilities	**0.612**	**0.000**	**0.671**	**0.000**	**0.114**	**0.068**
Distance to cultural facility	**−0.768**	**0.000**	**−0.603**	**0.000**	**−0.17**	**0.008**
Population density	0.468		0.50		0.475	
Commercial land use	**0.338**	**0.000**	**0.393**	**0.000**	0.026	0.669
**Travel behavior** *to explain the following exogenous observed variables*						
Active commuting	**0.432**	**0.000**			0.479	
PT commuting					**0.63**	**0.000**
Active NC travel	0.927		0.373	0.209		
PT travel			0.07			
*Chi-squared*	694.8		758.2		830.1	
*Goodness of fit index (GFI)*	0.849		0.875		0.839	

Note: figures in **bond** type mean they are statistically significant at the 1%, 5% or 10% level.

From the above results, we can make the following observations:

(1) As shown in Figure 4, the residential environment has direct and significant effects on travel behavior (including active travel behavior) for Cluster 1. Even though the above regression analyses confirmed that the residential environment had mixed effects on active travel, when the health-related QOL is treated as the final dependent variable when examining these effects, the effects of the residential environment on travel behavior disappear for Cluster 2.
(2) Figure 4 also reveals that different cause–effect relationships are derived with respect to the three clusters. Note that these three structures were uniquely derived based on a repeated trial-and-error process. In other words, no other alternative structures were found. However, in any cluster, it was found that travel behavior (including active travel behavior) does not have any influence on the health-related QOL in any direct or indirect ways. This finding is not consistent with the results of existing studies. In this study, frequency of travel by mode is introduced. In contrast, existing studies mostly just select the use of different modes. Even though walking and cycling contribute to the improvement of health in general, if the frequency is not high enough, the relevant effects on health may not be measurable. At the least, this case study supports the existence of such a possibility.
(3) For all three clusters, lifestyle habits have direct effects on the health-related QOL in a statistical sense. This is not surprising because it is not found for the first time since such effects on health have been confirmed widely in the field of public health. We have reconfirmed the same finding using a different set of data. In particular, the effects of lifestyle habits are most remarkable because the relevant total effects are the highest among all explanatory latent variables and all are statistically significant. Interestingly, for Cluster 1, it is confirmed that lifestyle habits have a significant effect on travel behavior.
(4) The residential environment has a direct effect on lifestyle habits for Clusters 2 and 3 (see Table 7), but as an overall effect, it does not affect the health-related QOL in this case study in Japan in either direct or indirect ways. As for factors characterizing the residential environment, population density is not relevant. This may suggest that, in Japan, further increasing the population density in residential areas is not beneficial to the final health outcome (*i.e*., QOL), even though it is widely recognized that emissions from car traffic will be reduced with the increase of population density as a result of the development of compact cities. Concerning other factors, both parks and cultural facilities are an important factor determining the quality of the residential environment for all three clusters, from the perspectives of both the distance from home and the number of parks and cultural facilities around the residential location. Commercial land use is relevant for influencing the health-related QOL in Clusters 1 and 2.
(5) While all the eight types of habits studied here are relevant to forming healthy lifestyle habits for Clusters 2 and 3, drinking alcohol is not important to people belonging to Cluster 1. As for the other six types of habits (breakfast, sleep, meals, not smoking, sports and working time), all are equally consistent and relevant for all the clusters. As regards drinking alcohol, we might associate the non-relevance of that habit with the type of lifestyle that we can observe from travel behavior, highly mobile and highly social, with frequent trips for shopping and leisure, for instance.
(6) Considering that most of the respondents in Cluster 2 can be associated with female gender, more advanced age, and bigger household size, the higher homogeneity in this latent construct may explain why this is not a valid or influential latent construct for the proposed structure in this cluster.
(7) As regards active travel, the relevance of commuting by active modes can be noted for Cluster 1, while the relevance of the use of public transport for commuting purposes can be noted for Cluster 3. For Cluster 2, no relevant factors associated with the noncommuting travel behavior (by active modes and by public transport) could be found.

**Table 7:** Totaleffects for the latent constructs.

Latent Constructs	Personal Attributes	Residential Environment	Travel Behavior	Lifestyle Habits
**Cluster 1**				
Travel behavior	**−0.350**	**0.297**		**0.240**
Lifestyle habits	0.008	0.035		
Health-related QOL	0.141	−0.042	−0.057	**0.480**
**Cluster 2**				
Lifestyle habits		**−0.233**	0.947	
Health-related QOL		−0.134	0.265	**0.280**
**Cluster 3**				
Travel behavior	**−0.415**	0.045		0.158
Lifestyle habits	**−0.162**	**−0.148**		
Health-related QOL	**0.114**	−0.08	−0.064	**0.626**

Note: figures in **bond** type mean they are statistically significant at the 1% or 5% level.

## 6. Conclusions

Body mass index (BMI), as a convenient measure of obesity, has been widely adopted to measure the health level in many existing studies. This indicator is to some extent suitable to explain the differences of physical health caused by residential environment in countries such as USA. However, it may not be the case in other countries. In Japan, clear associations between obesity, population density, and other environment features could not be clearly found in this study and other studies [48]. The health-related QOL approach can overcome the shortcomings of the BMI and provides more useful insights into policy making in the areas of urban design and planning in Japan. We found that commuters in Japanese cities have better scores in physical subscales (GH, PF) of the health-related QOL on one hand, while they have worse scores of mental subscales (VT, MH) than noncommuters.

However, we still recognize the necessity of including more consistent uses of transport and health measurement tools in future research, and the necessity of doing more collaborative work across the health, transport, and urban design sectors [32,60]. SF-36 appears to adequately explain the influences and direction of causality of the built environment and lifestyle habits and their effects on health-related QOL, but it appears to insufficiently explain the joint influence of the built environment and active travel behavior on the health-related QOL, even though the influences of the built environment on travel behavior and the influences of active travel behavior on health have been separately demonstrated. Additionally, it is still difficult to know with certitude the absolute and relative magnitudes of the true impacts of the built environment on travel behavior [52,53].

Some studies confirmed that neighborhood characteristics can be associated with individuals’ travel decisions, especially on nonmotorized travel frequency [51,61,62]. Similar findings can also be observed with respect to the influence of the built environment on the different types of active travel behavior that we employed in this study. A properly planned built environment contributes to a reduced dependency on cars and other private modes of transport due to the restrictions placed on their use, creating a necessity for greater use of public transportation and active travel modes—either as the way to reach a transit connection or to reach a final destination [63,64].

The complexity of the balance between the three dimensions of health remains high. We have observed how residential environments can induce different effects on the health-related QOL according to their own individual attributes and lifestyles, reflected here by travel frequency, by purpose, and by lifestyle habits. The results suggest that stress generated by commuting trips play a role in worsening the mental health conditions of urban dwellers, as we have been able to observe how noncommuters can enjoy greater vitality and better mental health conditions.

Some studies already suggest that commuting has some effects on happiness and anxiety, and it also affects day-to-day emotions more than overall evaluations of satisfaction with life or the sense that daily activities are worthwhile [65]. After all, commuting needs to be understood as an embodied and emotional practice that has implications for predicting travel behavior [66]. The conditions that might generate stress for Japanese commuters need to be examined in more detail in the future in health-related QOL research. On the other hand, commuters and active commuters will enjoy better physical functioning than the noncommuters. So, a specific travel behavior may lead to an improvement in one of the dimensions, while another dimension may worsen. For the social health components, no visible effects could be observed associated to the residential environment or travel behavior. This is in accordance with the ideas of other authors. For example, Ogilvie *et al.* stated that targeted behavior change programs can be effective in changing the transport choices of motivated subgroups, but the social distribution of their effects and their effects on the health of local populations are unclear [18].

For urban dwellers with less active and more sedentary lifestyles—that is, being less active in terms of movement than others—the proximity to facilities such as parks, supermarkets, and community centers was found to be much more important for all the dimensions of health. It would mean they have access to more services and opportunities for social contact within a reasonable distance in the neighborhood, a place where members of Cluster 2 and Cluster 3 spend more time of the day in comparison with residents in Cluster 1. For instance, as a part of the cultural facilities, the importance of community centers is understandable because of the many functions and services that these centers provide for the residents. The community centers help to promote health and the cultural and educational refinement of the citizens, to promote mutual connections and friendship within the community, and they allow citizens to engage in political activity and to otherwise participate, which are crucial to preventing social exclusion among the members of the community [55]. The results of the SEM model also highlight the relevance of the community centers and the mixed land use (commercial use included) to the residential environment.

We can easily assume that people who travel less and therefore spend more time at home might, for instance, be at higher risk of feeling lonely. Loneliness is a subjective, negative feeling related to the person’s own experience of deficient social relations. The determinants of loneliness are most often defined on external factors, which are absent in the social network, as the root of the loneliness [67]. Many people experience loneliness either as a result of living alone, a lack of close family ties, reduced connections with their culture of origin, or an inability to actively participate in the local community activities, and depression is a problem that often accompanies loneliness [67,68]. On the other hand, citizens with more active lifestyles are much less sensitive to the effects of distance from these facilities in terms of general health.

There is still an unexplored potential in understanding how the combination of urban design, land use patterns, and transportation systems can contribute to consolidating more active, healthier, and more livable communities [51,69].

Therefore, we recognize the need of new modeling approaches that can more comprehensively capture the complex causal relationships among health-related QOL, subjective well-being, travel behavior, and the residential environment. Many questions still remain about the interconnectedness of the built environment, travel behavior, and health [51,52,70] and should be considered for future research. We need to understand more about the environmental conditions that make urban dwellers be mobile and have a more active life, not only physically but also socially. The influence of lifestyle and health habits on active travel behavior has also been identified as a field where future research efforts should be focused [71].

In this study we found associations between the residential environment and the health-related QOL, but it is important to note that the former does not directly influence the latter. Rather, the residential environment imposes its influence *via* health promotion activities and lifestyle habits—including but not limited to travel behavior or active travel behavior [45,67].

A higher density of cultural facilities will encourage noncommuting travels in general for the members of Cluster 1, but interestingly and at the same time, a neighborhood with fewer cultural facilities in the surrounding area will mean that cycling activities will increase for members of Clusters 1 and 2. Population density is a key factor in promoting the numbers of active travel and noncommuting trips for all members in the sample, and in particular, results in more walking by commuters (Clusters 1 and 3) and more cycling by noncommuters (members of Cluster 2). Living in areas of greater density makes commuters of Cluster 3 more likely to consider commuting by active modes.

A mixed land use will influence positively the active travel commuting for respondents in Clusters 1 and 3, *i.e*., the commuters, as well as the active travel for noncommuting activities for all the respondents in the sample, particularly the walking behavior of individuals in Clusters 2 and 3, who are less likely to walk. This finding is totally consistent with the findings of other studies, that indicators associated with urban containment such as shorter distances to central services and facilities (and the subsequent reduction in travel times), and mixed land use, are all associated with less transit use, more walking, and active transport options [38,72,73,74].

From the SEM models, we observed how the health-related QOL is influenced by the residential environment via the lifestyle habits for the groups involving commuters, while the active travel behavior is mostly influenced by the individual attributes in the model, without being globally influenced by factors related to the residential environment, which have limited effects in particular groups. In case of noncommuters, it is found that neither travel behavior nor the residential environment affects the health-related QOL (precisely speaking, the self-reported health). Concerning the aforementioned insignificant influences of travel behavior on the health, one reason might be because the current health-related QOL does not have specific measures directly related to daily trip making (commuting and/or non-commuting). For example, a negative correlation between long-distance commuting and female fertility has been observed in Germany [75]. This result may imply that the SF-36 measurement is not actually suitable to reflect the impacts of daily travel on the health. Furthermore, there is very strong scientific evidence, based on a wide range of well-conducted studies in the USA, that physically active people have a lower risk profile for developing a number of disabling medical conditions and lower rates of various chronic diseases than do people who are inactive [76]. This may suggest that the self-reported health indicators may not be sufficient to capture the impacts of repeated daily travel on health. Considering that multitasking during use of public transportation systems and the liking of specific travel modes (e.g., car, bus, train, bicycle or walk) might be associated with positive utility of travel, these should be reflected in the conceptualization of travel behavior in future for deriving more sound conclusions.
